# The effect of dexmedetomidine on expression of neuronal nitric oxide synthase in spinal dorsal cord in a rat model with chronic neuropathic pain

**DOI:** 10.1055/s-0043-1761491

**Published:** 2023-04-14

**Authors:** Jun Pang, Suming Zhang, Ying Kong, Zhe Wang, Ruomeng Pei, Ping Zhuang, Xiaopeng Wang

**Affiliations:** 1 Tongji Shanxi Hospital, Third Hospital of Shanxi Medical University, Shanxi Academy of Medical Sciences, Shanxi Bethune Hospital, Department of Anesthesiology, Taiyuan, People's Republic of China.; 2 Huazhong University of Science and Technology, Tongji Hospital, Tongji Medical College, Wuhan, People's Republic of China.; 3 Xuzhou Medical University, The Affiliated Hospital of Xuzhou, Department of Critical Care Medicine, Xuzhou, People's Republic of China.

**Keywords:** Dexmedetomidine, Neuralgia, Nitric Oxide Synthase Type I, Dexmedetomidina, Neuralgia, Óxido Nítrico Sintase Tipo I

## Abstract

**Background** Neuropathic pain typically refers to the pain caused by somatosensory system injury or diseases, which is usually characterized by ambulatory pain, allodynia, and hyperalgesia. Nitric oxide produced by neuronal nitric oxide synthase (nNOS) in the spinal dorsal cord might serve a predominant role in regulating the algesia of neuropathic pain. The high efficacy and safety, as well as the plausible ability in providing comfort, entitle dexmedetomidine (DEX) to an effective anesthetic adjuvant. The aim of this study was to investigate the effect of DEX on the expression of nNOS in spinal dorsal cord in a rat model with chronic neuropathic pain.

**Methods** Male Sprague Dawley (SD) rats were randomly assigned into three groups: sham operation group (sham), (of the sciatic nerve) operation (CCI) group, and dexmedetomidine (DEX) group. Chronic neuropathic pain models in the CCI and DEX groups were established by sciatic nerve ligation. The thermal withdrawal latency (TWL) was measured on day 1 before operation and on day 1, 3, 7 and 14 after operation. Six animals were sacrificed after TWL measurement on day 7, and 14 days after operation, in each group, the L4–6 segment of the spinal cords was extracted for determination of nNOS expression by immunohistochemistry.

**Results** Compared with the sham group, the TWL threshold was significantly decreased and the expression of nNOS was up-regulated after operation in the CCI and DEX groups. Compared with the CCI grou[, the TWL threshold was significantly increased and the expression of nNOS was significantly down-regulated on day 7 and 14 days after operation in the DEX group.

**Conclusion** Down-regulated nNOS in the spinal dorsal cord is involved in the attenuation of neuropathic pain by DEX.

## Resumo

**Antecedentes** A dor neuropática refere-se tipicamente à dor causada por lesões ou doenças do sistema somatossensorial. De modo geral, é caracterizada por dor à ambulação, alodinia e hiperalgesia. O óxido nítrico produzido pela enzima óxido nítrico sintase neuronal (nNOS) na medula espinhal dorsal pode ter um papel predominante na regulação da dor neuropática. A alta eficácia e segurança, bem como a plausível capacidade de proporcionar conforto, faz com que a dexmedetomidina (DEX) seja um adjuvante anestésico eficaz. O objetivo deste estudo foi investigar o efeito da DEX na expressão de nNOS na medula espinhal dorsal em um modelo de ratos com dor neuropática crônica.

**Métodos** Ratos Sprague Dawley (SD) machos foram distribuídos aleatoriamente em três grupos: grupo de cirurgia simulada ( *sham* ), grupo de cirurgia (do nervo ciático; CCI) e grupo dexmedetomidina (DEX). Os modelos de dor neuropática crônica nos grupos CCI e DEX foram estabelecidos por ligadura do nervo ciático. A latência de retirada térmica (TWL) foi medida no dia 1 antes da cirurgia e nos dias 1, 3, 7 e 14 após o procedimento. Seis animais de cada grupo foram eutanasiados após a medida de TWL nos dias 7 e 14 após a cirurgia e o segmento L4-6 da medula espinhal foi extraído para determinação da expressão de nNOS por imuno-histoquímica.

**Resultados** Em comparação ao grupo *sham* , o limiar de TWL diminuiu significativamente e a expressão de nNOS foi regulada de maneira positiva após a cirurgia nos grupos CCI e DEX. Comparado ao grupo CCI, o limiar de TWL aumentou de forma significativa e a expressão de nNOS caiu significativamente diminuída nos dia 7 e 14 após a cirurgia no grupo DEX.

**Conclusão** A regulação negativa de nNOS na medula espinhal dorsal está envolvida na atenuação da dor neuropática pela DEX.

## INTRODUCTION

Neuropathic pain typically refers to the pain caused by somatosensory system injury or diseases, which is usually characterized by ambulatory pain, allodynia, and hyperalgesia. 1 The reported therapy methods (like pregabalin, gabapentin, and opiates, etc.) turn out to be of moderate efficiency, which largely hampers the quality of life of the involved patients and remains a challenging topic in clinic. 2 A previous study showed that nitric oxide (NO) produced by neuronal nitric oxide synthase (nNOS) in the spinal dorsal cord might serve a predominant role in regulating the algesia of neuropathic pain. 3 Specifically, peripheral spinal nerve injury can enhance the excitability of the spinal cord neuron by virtue of activating algesia in the afferent nerve, upon which the acceptor of N-methyl-D-aspartate was activated to produce NO in the spinal cord. 4 However, it was noticed that the effects of spinal NO on pain disorder or hypalgesia triggered by nerve injury are conflicting. 3

On the other hand, dexmedetomidine (DEX) refers to a highly-selective α-2 adrenoceptor (α2-AR) agonist, which shows analgesic effect when acting on C-type primary afferent fibers and α2-AR of the spinal dorsal cord, thus being therapeutically efficient for acute inflammatory, 5 postoperative, and neuropathic pain, 7 which is not sensitive toward opiates. Dexmedetomidine is characterized by the combined merits of sedation, analgesia, ability to suppress the sympathetic nerve and to enhance the postoperative recognition, etc. Its high efficacy and safety, as well as the plausible ability in providing comfort, make DEX an effective anesthetic adjuvant, which finds broad application in clinic. 8 9 10 A previous study evidenced that continuous intraperitoneal injection of DEX (40 μg·kg ^−1^ ) helped to alleviate neuropathic pain without triggering adverse reactions, the involved rats only showed mild lethargy. 11 Dexmedetomidine was also found to be capable of decreasing the use of propofol although the administration failed to improve bowel function. 12 The peripheral and central analgesia mechanisms involving suppressed transmission of pain signals, and the local analgesia mechanism in which hypalgesia was regulated by stimulating the α2 acceptor are possible for DEX. Despite all these facts, which can provide fundamental supports for the application of DEX in neuropathic pain therapy, the inherent analgesia mechanism remains ambiguous and awaits further exploration.

Taking together, the chronic constrictive injury (CCI) model was established in this study and subjected to continuous intraperitoneal injection of DEX, upon which the ethology variation and nNOS expression of the spinal dorsal cord were evaluated. The combined findings are expected to probe the analgesic effect of DEX on neuropathic pain and explore the related mechanisms.

## METHODS

### Animals

Male Sprague Dawley (SD) rats weighing 200 to 500 g from the Laboratory Animal Center of Xuzhou Medical University were used in this study. The study has been approved by the ethics committee of Shanxi Bethune Hospital (No. L20140226071).

### Reagents and instruments

The nNOS monoclonal rabbit antibodies were obtained from EPITOMICS, Inc (Burlingame, CA, USA). Polink-2 plusPolymer HRP Detection System (Rabbit) and concentrated DAB chromogenic kit were from (Zhongshan Golden Bridge Biotechnology Co., Ltd., Beijing, China) . Dexmedetomidine hydrochloride injection was obtained from Jiangsu Hengrui Pharmaceuticals Co., Ltd (Lianyungang, Jiangsu Province, China). The BM3–410A thermal radiation stimulator was from the Institute of Biomedical Engineering, Chinese Academy of Medical Sciences. Cryostat was purchased from Leica Biosystems Inc (Wetzlar, Germany).

### Animal group

A total of 36 male SD rats were randomly divided into three groups: sham operation group (sham), chronic constrictive injury (of sciatic nerve) operation group (CCI), and dexmedetomidine group (DEX). There were 12 rats in each group. Chronic neuropathic pain models in the CCI and DEX groups were established by sciatic nerve ligation, which was only isolated without ligation in the sham group. Intraperitoneal injection of 40μg·kg ^−1^ of DEX was conducted daily starting from the end of operation in the DEX group, while an equal volume of normal saline was injected in the sham and CCI groups.

### CCI model establishment

The CCI model was established based on the method proposed by Bennett. 13 Specifically, 10% chloral hydrate (300 mg·kg ^−1^ ) was intraperitoneally injected to afford anesthesia, upon which a longitudinal incision was made in the rear part of the left trochanter of rats to perform blunt dissection. The sciatic nerve trunk was exposed and periodically ligated with four strands using 4–0 silk thread with 1-mm intervals. The applicable strength followed the standard: calf muscles with slight tremor but exerting no impact on the blood supply of the sciatic nerve. Finally, sterile normal saline was utilized for rinsing, followed by layer-by-layer suturing. In contrast, the sciatic nerve was isolated and exposed but not subjected to ligation in the sham group.

### Determination of TWL

Thermal withdrawal latency (TWL) values were determined on day 1 before operation and on days 1, 3, 7, and 14 after operation. The Perspex box was placed onto a glass plate (thickness: 3 mm), the rats in the box were allowed to perform free activities for 30 minutes to accommodate the surrounding environment and temperature. Room temperature was maintained at 26°C ± 1°C. The method proposed by Hargreaves et al. was adopted to determine TWL. 14 The rat's paw on the operated side was irradiated with thermal radiometer. Thermal withdrawal latency value was measured and defined as the time interval ranging from irradiation starting time to the point when the paw was withdrawn. The irradiation intensity was kept consistent during this process. The upper limit for irradiation time was set as 25 seconds to avoid potential paw tissue injury. Five repeats with an interval of 10 minutes were adopted to finally calculate the average values.

### Determination of nNOS expression level of spinal dorsal cord

The expression of nNOS in the spinal dorsal horn was evaluated by immunohistochemistry. Intraperitoneal injection of 10% chloral hydrate (300 mg·kg ^−1^ ) was conducted to afford anesthesia for left ventricular puncture operation, followed by successive perfusion with sterile normal saline and paraformaldehyde (200 mL each). Intumescentia lumbalis (L _4–6_ ) of the spinal dorsal cord was extracted after the tissue turned stiff, which was stabilized in 4% paraformaldehyde overnight at 4°C. The tissue was put into 30% sucrose to initiate dehydration and precipitation. Serial frozen section was performed, with thickness of 30 μm. Subsequently, 0.01 mol L ^−1^ phosphate buffered saline (PBS) buffer was used to completely rinse the section, which was incubated for 30 minutes at 37°C using 3% H _2_ O _2_ -containing deionized water. Primary nNOS antibody was added after complete rinsing with PBS buffer to initiate the incubation for 36 hours at 4°C. The uncombined antibody was removed by washing with PBS buffer. Immunohistochemical working solutions were added in sequence for incubation, upon which the sections were then rinsed with PBS buffer and finally subjected to coloration for 5 minutes using diaminobenzidine (DAB) solution. Double-distilled water was utilized to rinse the sections, which were then firmly attached on anti-escaping slices and air dried. Gradient dehydration was performed with ethanol (75%, 95%, and 100%, 10 minutes each). The sections were then transparentized with xylene, sealed with neutral balsam, and observed as well as recorded by light microscope. The average optical density of nNOS on the spinal dorsal horn in the tissue pictures was statistically analyzed with the Image-Pro Plus 6.0 software (Media Cybernetics Inc., Rockville, MD, USA).

### Statistical analysis

The SPSS Statistics for Windows, version 16.0 software (SPSS Inc., Chicago, IL, USA) was used for statistical analysis in this study. All the data were represented by mean ± standard deviation (X ± SD); the repeatedly measured data were subjected to analysis of variance (ANOVA) for repeated measurement, while the comparison among the groups adopted one-way ANOVA, with *p* < 0.05 indicating that the difference was of statistical significance and *p* < 0.01 denoting highly significant difference.

## RESULTS

### TWL values of the involved rats at different time intervals

**Figure 1:**
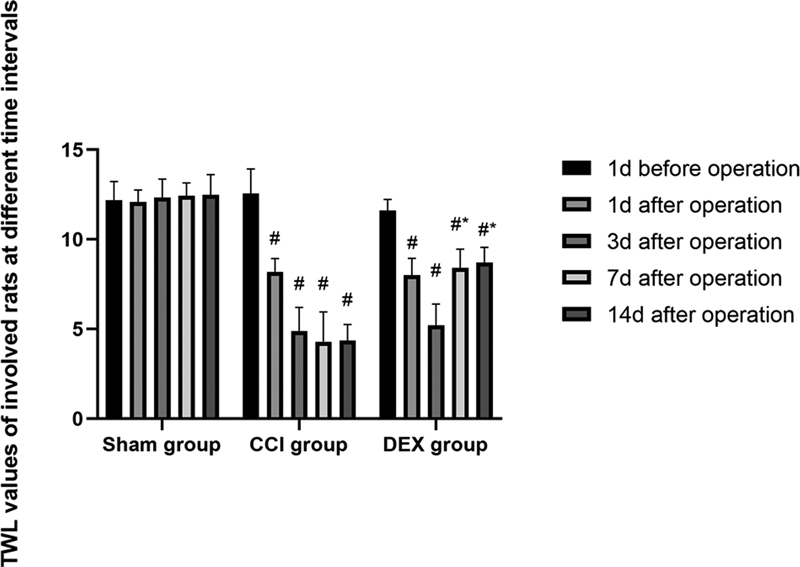
Thermal withdrawal latency values of involved rats at different time intervals. **Notes:** ^#^ represents *P* < 0.05, which was compared with that of Sham group; * represents *P* < 0.05, which was compared with that of CCI group.

The rats in the CCI and DEX groups after operation were characterized by hyperalgesia symptoms, with paw adduction on the operated side and autophagy as well as claudication, which were, however, absent in the sham group. The repeated measures ANOVA suggested that the difference of TWL values at different time points (day) was of statistical significance (F = 69.17, *p* < 0.05); the difference among different groups was also of statistical significance (F = 273.90, *p* < 0.05). The interaction among the groups at different time points was also identified as statistically significant (F = 30.90, *p* < 0.05). The combined results suggest that the TWL variation tendency varies substantially among these groups. Relative to sham group, the TWL values of rats after operation experienced dramatic decrease in the CCI and DEX groups ( *p* < 0.05); compared with the CCI group, the TWL increased remarkably for the rats 7 and 14 days after operation in the DEX group ( *p* < 0.05) ( Figure 1 ).

### nNOS expression of spinal dorsal cord

**Figure 2:**
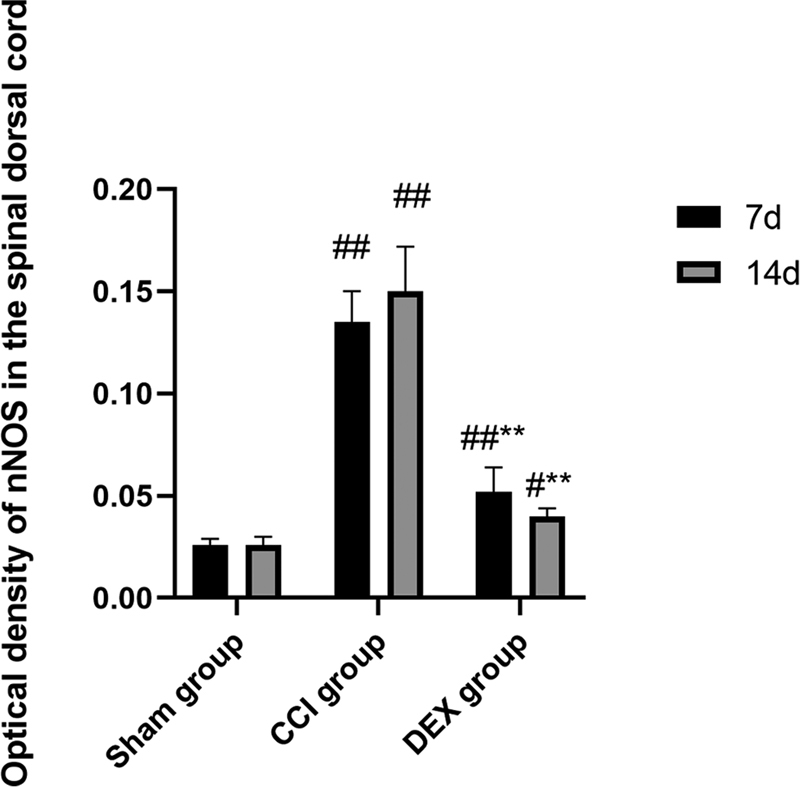
Mean optical density of neuronal nitric oxide synthase (nNOS) in the spinal dorsal cord of rats. Notes: ## represents *P* < 0.01 and # represents *P* < 0.05, which were compared with that of sham group; ** represents *P* < 0.01, which was compared with that of chronic constrictive injury group.

**Figure 3:**
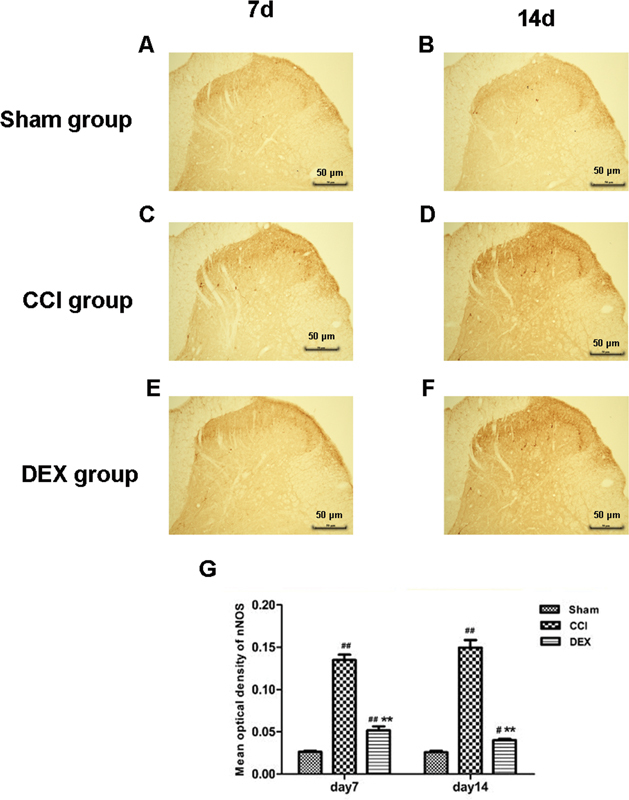
Neuronal nitric oxide synthase expression of the spinal dorsal cord. The expression of nNOS in spinal dorsal horn was evaluated by immunohistochemistry. Neuronal nitric oxide synthase expression of spinal dorsal cord of rats in the three groups: nNOS expression of spinal dorsal cord in sham group (A) 7 and (B) 14 days after operation; nNOS expression of spinal dorsal cord in chronic constrictive injury (CCI) group (C) 7 and (D) 14 days after operation; nNOS expression of spinal dorsal cord in DEX group (E) 7 and (F) 14 days after operation; (G) Plot of mean optical density of nNOS of rats 7 and 14 days after operation in the three groups. Scale bar: 50 μm. Notes: ## represents *P* < 0.01, which were compared with that of Sham group; ** represents *P* < 0.01, which was compared with that of CCI group. Six rats have been evaluated in each group.

Compared with the sham group, the measured mean optical density values of nNOS in the spinal dorsal cord of rats in both CCI group were evidently higher for either 7 or 14 days after the operation, and the differences were highly significant ( *p* < 0.05), as clearly shown in Figure 2 . Similarly, the values in the CCI group 7 days after the operation also show significant difference when compared with those of the sham group ( *p* < 0.01), but the difference turned to be less at 14 days after operation ( *p* < 0.05). Notably, compared with the CCI group, the values in the DEX group show highly significant differences for 7 or 14 days after the operation ( *p* < 0.01). It is acknowledged that the positive reaction of the immunohistochemistry method is characterized by brown granules, which primarily express in the neuronal cytoplasm of lamina II in the spinal dorsal cord. The changes of mean optical density among the three groups are also reflected in Figure 3 . The combined results suggest that compared with the sham group, nNOS expression of the spinal dorsal cord within the operated side increased in both CCI and DEX groups. Compared with the CCI group, continuous intraperitoneal injection gave rise to the deceased nNOs in the lamina II of the spinal dorsal cord of rats 7 and 14 days after the operation in the DEX group, and the differences were also of statistical significance ( *p* < 0.01).

## DISCUSSION

The spinal cord serves as the relay station for the delivery of pain messages from the peripheral region to the brain. Myelinated Aδ fiber with medium diameter and nonmyelinated C fiber with small diameter terminate at the lamina superficialis of the spinal dorsal cord, the two fibers mediate the delivery of nociceptive information. To this end, the lamina superficialis has generally been recognized as the position relating to pain delivery. Endogenous nitric oxygen (NO) functions as an important neurotransmitter in the central nervous system. The detailed mechanisms for precise regulation of the generation, release, diffusion, and inactivation of NO exist within the nervous system, which are primarily performed by regulating the nNOS activity. It is well documented that NO participates in tuning algesia at different levels mediated by periphery and center during nociceptive information delivery, by virtue of enhancing neuron excitability and canceling the descending suppression toward the spinal cord. That is, NO exerts significant impact on the processing of algesia signal, which stands out especially at the spinal level. It is, however, noticed that the increased nNOS activity may lead to the overproduction of NO, which further weakens vasodilatation and causes hypotension. 15 Nitric oxide also tends to suppress the activity of antioxidant enzyme and, therefore, increase the oxidative pressure. 16 As a free radical, the overproduction of NO will suppress oxidative phosphorylation and reduce oxygen consumption. 17 Nitric oxide is also likely to interact with other reactive oxygen species and form active components with stronger toxicity, thus damaging DNA, protein, and cytomembrane, as well as increasing the permeability of mitochondria. 18

In this study, chronic neuropathic pain of the sciatic nerve in rat model was adopted to afford noxious stimulation triggered by the pain behavior. The combined results suggest that TWL decreased upon the ligation of the sciatic nerve, accompanied with increased expression of nNOS of the spinal dorsal cord. The mechanism is explained as follows: noxious stimulation contributed to the increased release of glutamic acid, which further activated the acceptor NMDA of the spinal dorsal cord and resulted in the flowing of a large amount of Ca ^2+^ into the neuron; then, nNOS was activated and characterized by up-regulated expression. 19 Dexmedetomidine constitutes a new highly-selective α2-AR agonist and exhibits abirritation effect when acting on C-type afferent fiber and spinal dorsal cord α2-AR. 20 Dexmedetomidine is also capable of central analgesia by depolarizing locus coeruleus and presynaptic membrane of noradrenergic access of spinal and suppressing the release of substance P and other pain peptides, upon which the pain stimuli can be suppressed and pain signaling can be terminated. In addition, local analgesia also takes effect by stimulating the α2 acceptor to regulate hypalgesia. 21 22

It was found that TWL increased significantly on the 7 ^th^ day after ligation of the sciatic nerve and continuous intraperitoneal injection in the DEX group, thereby signifying that neuropathic pain can be substantially alleviated via continuous intraperitoneal DEX injection. Dexmedetomidine can be characterized by its strong anti-nociceptive effect by directly suppressing the release of excitatory neurotransmitters (like glutamic acid) from the synaptic afferent terminus of the spinal dorsal cord. Therefore, the increased nNOS expression induced by DEX, which suppressed nerve injury, is probably completed by suppressing the release of pain-related neurotransmitter of primary sensory neuron. 23 24 The diverse analgesia pathways of DEX can be performed by intravenous or intrathecal administration to improve postoperative effect. Also, its wide application range and safety allow DEX to be applied in various postoperative analgesia cases. However, DEX also shows side effects even within the regular use range, such as bradycardia and hypotension, which are related to the administration dosage. In this regard, the dosage and administration method should be strictly controlled in clinic. 25

In conclusion, the above findings suggest that continuous intraperitoneal DEX injection can efficiently alleviate the hyperpathia of neuropathic pain, and the suppressed expression of nNOS in the spinal dorsal cord may serve as the potential mechanism in terms of alleviating the symptoms assigned to neuropathic pain.
