# Erratum

**DOI:** 10.1055/s-0043-1775834

**Published:** 2023-10-29

In the article “The effect of dexmedetomidine on expression of neuronal nitric oxide synthase in spinal dorsal cord in a rat model with chronic neuropathic pain,” with DOI code number https://doi.org/10.1055/s-0043-1761491 , published in the Arq Neuropsiquiatr. 2023; 81(03): 233–239:

The Figure 3F:

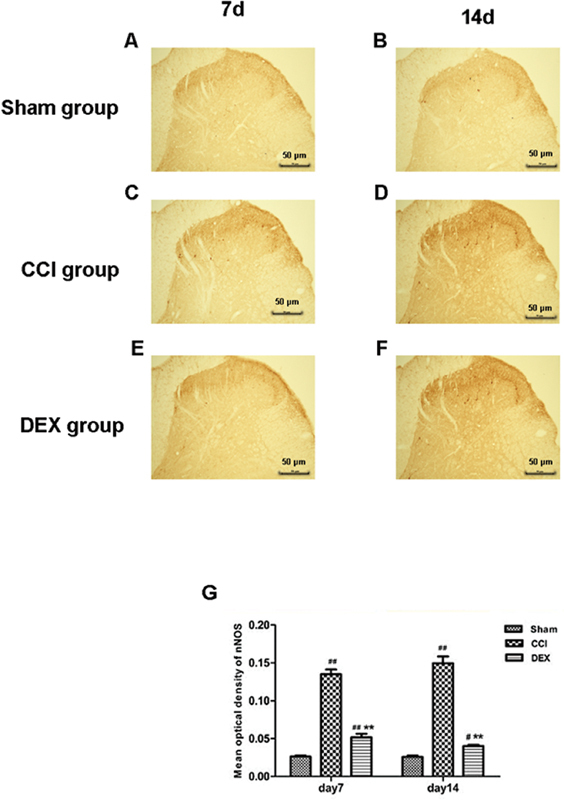



Should be:



